# Supplementary material for: Value of laboratory results in addition to vital signs in a machine learning algorithm to predict in-hospital cardiac arrest: A single-center retrospective cohort study
Source: PLoS One. 2020 Jul 13;15(7):e0235835. doi: 10.1371/journal.pone.0235835 (PMC7357766; doi:10.1371/journal.pone.0235835)
Supplement: S1 File — (DOCX) [file pone.0235835.s001.docx]

**Supplementary**

**Supplementary documents**

- List of data inconsistencies
- Details of Model Development
- Methods of sensitivity analyses
- Missing Imputation
- sTable 1: Vital Signs and laboratory results
- sTable2: Proportion of Missing Variables in the entire Dataset
- sTable3: Sensitivity Analyses with Various Missing Imputation Methods
- sFigure1: A Detailed Architectural Overview of Data Extraction and Representation

**Online Supplementary**

[https://github.com/liyuan9988/Automet](https://github.com/liyuan9988/Automet?fbclid=IwAR2xXzGwEbjfGqxwKPR_MonXNRzY4bMWAT22vSStl8JG6tEdrF03_nhErho)

**List of data inconsistencies**

- 1. body temperature <30℃ or >45℃
  2. heart rate <20/min or >300/min
  3. respiratory rate >80/min
  4. systolic blood pressure <20 mm Hg or >300 mm Hg
  5. diastolic blood pressure <10 mm Hg or >200 mm Hg
  6. urine output >10000ml per eight hour
  7. oxygen saturation <40% or >101%

**Detailed model development**

1. **Random Forest Model**
   - **﻿**Random forest is an ensemble of decision trees created by samples of the training data and random feature selection in tree induction
   - Each ‘tree’ will give a classification results and the ‘forest’ will return the probability of a certain event by calculating the number of ‘trees’ that predicts the event to occur.
2. **Bagging of the datasets**
   - To cope with the imbalance between the number of patients with IHCA and those without, we utilized the under-sampling bagging method. We trained one random forest model based on all IHCA-positive data and randomly sampled IHCA-negative data. We repeated this procedure 10 times. At test time, the prediction is made from the average of the prediction of those 10 random forest models.^[[1]](#footnote-1)^ (**sFigure 1**)
   - The training procedure involves the random selection of the dataset, which influences the performance of the model. Hence, we evaluate 100 models, each consists of 10 random forest models, which is trained based on the different under-sampled data.
   - For the test dataset, IHCA patients were mixed with randomly-selected non-IHCA patients at the proportion of 1:1000. (**sFigure 1**)
3. **Parameter setting of random forest model**

We have used scikit-learn^[[2]](#footnote-2)^ implementation of random forest model. The parameter was set to the default value except for the number of estimators, which was set to 100.

**Methods for sensitivity analyses**

- Medical/Surgical
  - Patients admitted to the department that does any types of surgery (i.e. general surgery, neurosurgery, cardiothoracic surgery, ophthalmology, ENT, obstetric and gynecology, urology, orthopedics, plastic surgery, breast surgery, and dermatology) were classified as surgery regardless of actually undergoing surgery.
  - Using the same methodology for our primary analysis, model derivation were done in a mixed cohort of medical and surgical patients. Validation was performed separately in each population.
- ICU/Ward
  - To account for the transfer between ICU and ward in a same patient, each patient was labeled as ICU patient if they have ever stayed in the ICU in the last 48 hours, otherwise patients were labeled as ward patient.
  - Model derivation and validation were done in the same approach as Medical/Surgical subgroup.

**Detailed methods of missing imputations**

- First, we have a priori prepared the following methods of missing handling methods.
  1. **[imputation]** Missing values were imputed with the patient’s last measured value for that feature or the median value of the entire sample if a patient had no previous values.
  2. **[binary conversion]** Convert the variable into binary (if the value wasmissing, converted to 0, otherwise 1.)
  3. **[categorical conversion]** The variable was converted into a categorical variable following the ANZIC-CORE rule.
  4. **[deletion]** The entire variable was excluded from the analysis
- We have combined those four methods according to the percentage of missing and repeated the primary analysis
  1. Regardless of the percentage of missing, use **[imputation]**
  2. If >50% was missing, use **[binary conversion]**, otherwise use **[imputation]**
  3. If >50% was missing, use **[categorical conversion]**, otherwise use **[imputation]**
  4. If >50% was missing, use **[deletion]**, otherwise use **[imputation]**

**sTable 1: Vital Signs and laboratory results**

|  | Training | | | | Test | | | |
| --- | --- | --- | --- | --- | --- | --- | --- | --- |
|  | IHCA | | non-IHCA | | IHCA | | non-IHCA | |
|  | mean | sd | mean | sd | mean | sd | mean | sd |
| Days since Admission | 23 | 52 | 27 | 49 | 28 | 58 | 24 | 39 |
| sBP_1 | 115 | 18 | 120 | 15 | 111 | 19 | 121 | 15 |
| sBP_2 | 115 | 19 | 120 | 16 | 112 | 19 | 121 | 16 |
| sBP_3 | 114 | 20 | 120 | 16 | 112 | 19 | 121 | 17 |
| sBP_4 | 114 | 22 | 120 | 17 | 111 | 21 | 121 | 17 |
| sBP_5 | 112 | 20 | 120 | 17 | 111 | 21 | 121 | 17 |
| sBP_6 | 112 | 21 | 120 | 17 | 108 | 23 | 121 | 17 |
| dBP_1 | 65 | 13 | 68 | 10 | 64 | 13 | 69 | 10 |
| dBP_2 | 63 | 14 | 68 | 11 | 63 | 14 | 69 | 11 |
| dBP_3 | 64 | 14 | 68 | 11 | 65 | 15 | 69 | 11 |
| dBP_4 | 63 | 13 | 68 | 11 | 63 | 15 | 69 | 11 |
| dBP_5 | 63 | 15 | 68 | 12 | 63 | 14 | 69 | 12 |
| dBP_6 | 61 | 15 | 68 | 12 | 62 | 15 | 69 | 12 |
| hr_1 | 85 | 16 | 78 | 12 | 87 | 20 | 78 | 12 |
| hr_2 | 86 | 19 | 78 | 13 | 88 | 20 | 78 | 13 |
| hr_3 | 85 | 20 | 78 | 13 | 88 | 21 | 78 | 13 |
| hr_4 | 88 | 19 | 78 | 13 | 89 | 22 | 77 | 14 |
| hr_5 | 88 | 21 | 77 | 14 | 91 | 23 | 77 | 14 |
| hr_6 | 90 | 23 | 77 | 14 | 95 | 25 | 77 | 14 |
| rr_1 | 0.6 | 0.5 | 0.9 | 0.3 | 0.6 | 0.5 | 0.8 | 0.4 |
| rr_2 | 0.6 | 0.5 | 0.9 | 0.3 | 0.6 | 0.5 | 0.8 | 0.4 |
| rr_3 | 0.6 | 0.5 | 0.9 | 0.3 | 0.5 | 0.5 | 0.8 | 0.4 |
| rr_4 | 0.6 | 0.5 | 0.9 | 0.3 | 0.5 | 0.5 | 0.8 | 0.4 |
| rr_5 | 0.6 | 0.5 | 0.9 | 0.3 | 0.5 | 0.5 | 0.8 | 0.4 |
| rr_6 | 0.5 | 0.5 | 0.9 | 0.3 | 0.4 | 0.5 | 0.8 | 0.4 |
| saturation_1 | 97.0 | 2.0 | 97.1 | 1.5 | 96.9 | 1.6 | 97.0 | 1.2 |
| saturation_2 | 97.1 | 1.9 | 97.2 | 1.6 | 96.6 | 4.1 | 97.0 | 1.5 |
| saturation_3 | 96.7 | 3.1 | 97.2 | 1.6 | 97.0 | 1.6 | 97.0 | 1.6 |
| saturation_4 | 96.8 | 3.3 | 97.2 | 1.7 | 97.0 | 1.6 | 97.0 | 1.5 |
| saturation_5 | 96.8 | 2.5 | 97.2 | 1.8 | 96.8 | 2.0 | 97.0 | 1.6 |
| saturation_6 | 96.6 | 3.5 | 97.2 | 1.7 | 96.0 | 4.0 | 97.1 | 1.4 |
| temp_1 | 36.4 | 0.8 | 36.3 | 0.5 | 36.6 | 0.8 | 36.4 | 0.5 |
| temp_2 | 36.5 | 0.9 | 36.3 | 0.5 | 36.7 | 0.8 | 36.3 | 0.5 |
| temp_3 | 36.6 | 0.9 | 36.3 | 0.5 | 36.6 | 0.8 | 36.3 | 0.5 |
| temp_4 | 36.6 | 0.9 | 36.3 | 0.5 | 36.6 | 0.8 | 36.3 | 0.5 |
| temp_5 | 36.6 | 0.9 | 36.3 | 0.5 | 36.7 | 0.8 | 36.3 | 0.5 |
| temp_6 | 36.5 | 0.9 | 36.3 | 0.6 | 36.8 | 1.1 | 36.3 | 0.5 |
| urine_1 | 0.4 | 0.5 | 0.8 | 0.4 | 0.6 | 0.5 | 0.8 | 0.4 |
| urine_2 | 0.4 | 0.5 | 0.8 | 0.4 | 0.5 | 0.5 | 0.8 | 0.4 |
| urine_3 | 0.4 | 0.5 | 0.8 | 0.4 | 0.5 | 0.5 | 0.8 | 0.4 |
| urine_4 | 0.4 | 0.5 | 0.8 | 0.4 | 0.5 | 0.5 | 0.8 | 0.4 |
| urine_5 | 0.4 | 0.5 | 0.8 | 0.4 | 0.5 | 0.5 | 0.8 | 0.4 |
| urine_6 | 0.4 | 0.5 | 0.8 | 0.4 | 0.4 | 0.5 | 0.8 | 0.4 |
| wbc_1 | 86 | 55 | 72 | 38 | 85 | 50 | 73 | 38 |
| wbc_2 | 93 | 60 | 72 | 42 | 88 | 47 | 72 | 38 |
| rbc_1 | 351 | 65 | 363 | 60 | 348 | 68 | 363 | 59 |
| rbc_2 | 350 | 70 | 364 | 62 | 348 | 78 | 365 | 62 |
| hb_1 | 11 | 2 | 11 | 2 | 10 | 2 | 11 | 2 |
| hb_2 | 11 | 2 | 11 | 2 | 10 | 2 | 11 | 2 |
| hct_1 | 32 | 5 | 33 | 5 | 32 | 6 | 33 | 5 |
| hct_2 | 32 | 6 | 33 | 5 | 32 | 7 | 33 | 5 |
| mcv_1 | 91 | 8 | 91 | 5 | 91 | 6 | 91 | 5 |
| mcv_2 | 91 | 8 | 91 | 5 | 91 | 6 | 91 | 5 |
| mch_1 | 30 | 3 | 30 | 2 | 30 | 2 | 30 | 2 |
| mch_2 | 30 | 3 | 30 | 2 | 30 | 2 | 30 | 2 |
| mchc_1 | 33 | 1 | 33 | 1 | 33 | 1 | 33 | 1 |
| mchc_2 | 33 | 2 | 33 | 1 | 33 | 1 | 33 | 1 |
| plt_1 | 18 | 8 | 21 | 9 | 18 | 11 | 22 | 10 |
| plt_2 | 17 | 9 | 21 | 9 | 18 | 11 | 22 | 10 |
| rdw_1 | 16 | 3 | 15 | 2 | 16 | 2 | 15 | 2 |
| rdw_2 | 16 | 3 | 15 | 2 | 16 | 2 | 15 | 2 |
| tp_1 | 6.0 | 0.8 | 6.1 | 0.6 | 6.0 | 0.9 | 6.1 | 0.6 |
| tp_2 | 5.9 | 0.9 | 6.1 | 0.7 | 6.0 | 0.9 | 6.1 | 0.6 |
| alb_1 | 2.8 | 0.6 | 3.1 | 0.5 | 2.9 | 0.7 | 3.1 | 0.5 |
| alb_2 | 2.8 | 0.7 | 3.1 | 0.6 | 2.9 | 0.7 | 3.1 | 0.6 |
| ast_1 | 98 | 270 | 38 | 174 | 104 | 385 | 38 | 81 |
| ast_2 | 135 | 377 | 36 | 61 | 133 | 440 | 37 | 74 |
| alt_1 | 79 | 184 | 34 | 75 | 72 | 187 | 35 | 70 |
| alt_2 | 98 | 244 | 33 | 53 | 90 | 232 | 35 | 66 |
| ld_1 | 397 | 477 | 254 | 211 | 414 | 602 | 250 | 195 |
| ld_2 | 461 | 576 | 251 | 193 | 450 | 619 | 248 | 196 |
| ALP_1 | 344 | 211 | 332 | 304 | 385 | 370 | 334 | 265 |
| ALP_2 | 335 | 200 | 330 | 313 | 384 | 379 | 330 | 267 |
| gGTP_1 | 71 | 66 | 77 | 108 | 105 | 154 | 80 | 113 |
| gGTP_2 | 70 | 67 | 76 | 111 | 104 | 158 | 79 | 117 |
| t_bil_1 | 1.0 | 1.4 | 0.8 | 0.9 | 1.2 | 1.6 | 0.9 | 1.2 |
| t_bil_2 | 1.1 | 1.7 | 0.8 | 1.0 | 1.2 | 1.8 | 0.9 | 1.2 |
| i_bil_1 | 0.8 | 0.4 | 0.9 | 0.4 | 0.8 | 0.4 | 0.9 | 0.3 |
| i_bil_2 | 0.7 | 0.4 | 0.9 | 0.4 | 0.7 | 0.4 | 0.9 | 0.3 |
| ck_1 | 0.5 | 0.5 | 0.8 | 0.4 | 0.6 | 0.5 | 0.8 | 0.4 |
| ck_2 | 0.5 | 0.5 | 0.8 | 0.4 | 0.6 | 0.5 | 0.8 | 0.4 |
| ck_mb_1 | 0.9 | 0.3 | 1.0 | 0.1 | 0.9 | 0.3 | 1.0 | 0.1 |
| ck_mb_2 | 0.8 | 0.4 | 1.0 | 0.1 | 0.8 | 0.4 | 1.0 | 0.1 |
| bun_1 | 35 | 22 | 20 | 12 | 32 | 23 | 20 | 12 |
| bun_2 | 36 | 22 | 20 | 13 | 34 | 23 | 20 | 12 |
| cre_1 | 2.1 | 2.3 | 1.2 | 1.3 | 2.0 | 2.2 | 1.2 | 1.4 |
| cre_2 | 2.2 | 2.2 | 1.2 | 1.3 | 2.1 | 2.3 | 1.2 | 1.4 |
| eGFR_1 | 51 | 38 | 72 | 33 | 63 | 57 | 71 | 30 |
| eGFR_2 | 49 | 38 | 72 | 33 | 58 | 58 | 71 | 31 |
| sodium_1 | 139 | 6 | 139 | 3 | 139 | 5 | 139 | 3 |
| sodium_2 | 139 | 7 | 139 | 3 | 139 | 6 | 139 | 3 |
| potassium_1 | 4.3 | 0.7 | 4.1 | 0.4 | 4.1 | 0.6 | 4.1 | 0.4 |
| potassium_2 | 4.3 | 0.8 | 4.1 | 0.4 | 4.2 | 0.6 | 4.1 | 0.4 |
| chrol_1 | 103 | 7 | 104 | 4 | 103 | 6 | 104 | 4 |
| chrol_2 | 103 | 7 | 104 | 4 | 103 | 6 | 104 | 4 |
| glu_1 | 0.8 | 0.4 | 0.9 | 0.3 | 0.8 | 0.4 | 0.9 | 0.3 |
| glu_2 | 0.7 | 0.4 | 0.9 | 0.3 | 0.8 | 0.4 | 0.9 | 0.3 |
| crp_1 | 6.3 | 7.2 | 3.2 | 3.5 | 5.4 | 6.7 | 3.4 | 3.8 |
| crp_2 | 6.6 | 7.2 | 3.2 | 3.6 | 5.9 | 7.2 | 3.3 | 3.8 |
| calcium_1 | 0.7 | 0.5 | 0.9 | 0.3 | 0.8 | 0.4 | 0.9 | 0.3 |
| calcium_2 | 0.6 | 0.5 | 0.9 | 0.3 | 0.7 | 0.4 | 0.9 | 0.3 |
| magnesium_1 | 0.8 | 0.4 | 0.9 | 0.3 | 0.7 | 0.4 | 0.9 | 0.3 |
| magnesium_2 | 0.7 | 0.5 | 0.9 | 0.3 | 0.8 | 0.4 | 0.9 | 0.3 |
| bnp_1 | 0.7 | 0.5 | 0.9 | 0.3 | 0.8 | 0.4 | 0.9 | 0.2 |
| bnp_2 | 0.6 | 0.5 | 0.9 | 0.3 | 0.8 | 0.4 | 0.9 | 0.2 |
| pt_inr_1 | 1.4 | 0.8 | 1.3 | 0.3 | 1.4 | 0.5 | 1.2 | 0.3 |
| pt_inr_2 | 1.5 | 1.0 | 1.3 | 0.3 | 1.5 | 1.0 | 1.2 | 0.3 |
| pt_1 | 70 | 23 | 80 | 16 | 74 | 24 | 86 | 16 |
| pt_2 | 68 | 24 | 81 | 17 | 70 | 26 | 87 | 16 |
| aptt_1 | 43 | 25 | 35 | 8 | 45 | 27 | 36 | 8 |
| aptt_2 | 45 | 28 | 35 | 8 | 47 | 26 | 36 | 7 |

Each digit after each variable name denotes the sequence each variable was measured. Larger number means newer results.

Abbreviations: alb, albumin; aptt, activated partial thromboplastin time; ast, aspartate aminotransferase; alt, alanine aminotransferase; ALP, alkaline phosphatase; bnp, brain natriuretic peptide; CK, creatine kinase; CK-MB, creatine kinase – muscle/brain; Cre, creatinine; crp, C-reactive protein; dBP, diastolic blood pressure; eGFR, estimated estimated Glomerular. Filtration Rate; gGTP, gamma-glutamyltransferase; glu, glucose, hb, hemoglobin; hct, hematocrit; hr, heart rate; i_bil, indirect bilirubin; ldh, lactate dehydrogenase; mch, mean corpuscular hemoglobin; mchc, mean corpuscular haemoglobin concentration; mcv, mean corpuscular volume; plt, platelets; rdw, red cell distribution width; pt, prothrombin time; pt_inr, prothrombin time international normalized ratio; rbc, red blood cells; rr, respiratory rate, sbp, systolic blood pressure; temp, body temperature; tp, total protein;t_bil, total bilirubin; urine, urine output; wbc, white blood cells

**sTable2: Proportion of Missing Variables in the entire Dataset**

| **variable** | **Missing (%)** |
| --- | --- |
| **Blood Pressure** | 21 |
| **Heart Rate** | 15 |
| **Respiratory Rate*** | 80 |
| **Urine Output*** | 72 |
| **Saturation** | 36 |
| **Temperature** | 17 |
| **WBC** | 15 |
| **RBC** | 15 |
| **Hb** | 15 |
| **Hct** | 15 |
| **MCV** | 15 |
| **MCH** | 15 |
| **MCHC** | 15 |
| **Plt** | 15 |
| **RDW** | 17 |
| **TP** | 47 |
| **Alb** | 46 |
| **AST** | 36 |
| **ALT** | 36 |
| **LD** | 42 |
| **ALP** | 50 |
| **GGT** | 49 |
| **Total Bilirubin** | 38 |
| **Indirect bilirubin*** | 64 |
| **CK*** | 52 |
| **CK-MB*** | 94 |
| **BUN** | 36 |
| **Cre** | 35 |
| **eGFR** | 41 |
| **Sodium** | 35 |
| **Potassium** | 35 |
| **Chloride** | 36 |
| **Glucose*** | 69 |
| **CRP** | 43 |
| **Calcium*** | 72 |
| **Magnesium*** | 83 |
| **BNP*** | 86 |
| **PT-INR** | 42 |
| **PT** | 42 |
| **APTT** | 43 |

*if more than half of a variable was missing, it was converted to binary variable. (i.e. 0 if missing; 1 if measured)

Abbreviations:

Alb, albumin; APTT, activated partial thromboplastin time; AST, aspartate aminotransferase; ALT, alanine aminotransferase; ALP, alkaline phosphatase; BNP, brain natriuretic peptide; CK, creatine kinase; CK-MB, creatine kinase – muscle/brain; Cre, creatinine; CRP, C-reactive protein; eGFR, estimated estimated Glomerular. Filtration Rate; GGT, gamma-glutamyltransferase; Hb, hemoglobin; Hct, hematocrit; LDH, lactate dehydrogenase; MCH, mean corpuscular hemoglobin; MCHC, mean corpuscular haemoglobin concentration; MCV, mean corpuscular volume; Plt, platelets; RDW, red cell distribution width; PT, prothrombin time; PT-INR, prothrombin time international normalized ratio; RBC, red blood cells; TP, total protein; WBC, white blood cells

**Supplementary Table 3: Predictive Performance of Each Model for the In-hospital Cardiac Arrest with Various Imputation Methods**

|  | Imputation methods | | | |
| --- | --- | --- | --- | --- |
| IHCA | Imputation | Binary | Deletion | Categorical |
| 0-8h Vitals-Only | 0.877 | 0.896 | 0.887 | 0.899 |
| 0-8h Vitals+Labs | 0.855 | 0.864 | 0.848 | 0.862 |
| 0-24h Vitals-Only | 0.851 | 0.871 | 0.850 | 0.873 |
| 0-24h Viltas+Labs | 0.828 | 0.849 | 0.828 | 0.846 |

Missing values were imputed with the most recent variable value or average of the overall sample if <50% of the values were missing. Otherwise, a variable was converted according to the following rules:

1) Imputation (the variable was imputed with the most recent variable value or average of the overall sample);

2) Binary (if the variable was missing it was converted to 0, otherwise it was set to 1);

3) Categorical (the variable was converted to a categorical value, i.e. missing or quantile category);

4) Deletion (the variable was removed from the entire analysis).

Please refer to the **Missing Imputation** section of this document for further details of the missing imputation methods.

**sFigure 1: A Detailed Architectural Overview of Data Extraction and Representation

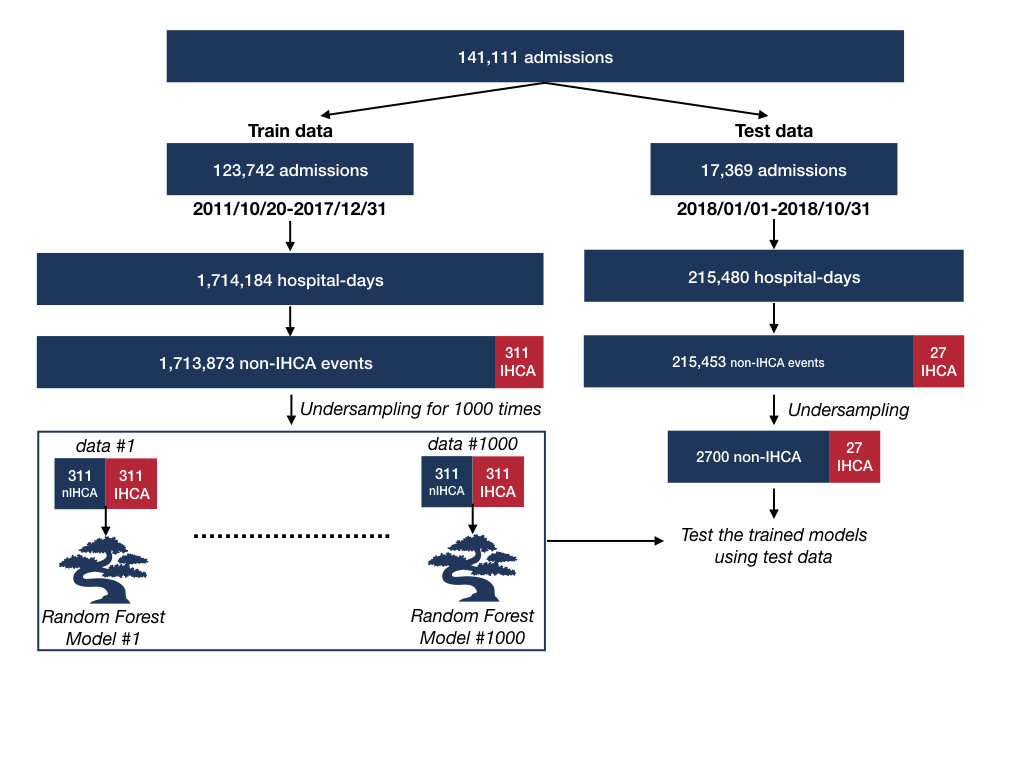
**

1. Barandela, R., Valdovinos, R. & Sánchez, New Applications of Ensembles of Classifiers,

   J. *Patt. Analy. App*. (2003) 6: 245. https://doi.org/10.1007/s10044-003-0192-z [↑](#footnote-ref-1)
2. Pedregosa *et al.*, [Scikit-learn: Machine Learning in Python](http://jmlr.csail.mit.edu/papers/v12/pedregosa11a.html), JMLR 12, pp. 2825-2830, 2011. [↑](#footnote-ref-2)
